# The role of Galacto-oligosaccharides (GOS) in the recovery from dysbiosis in patients on long-term atypical antipsychotic treatment

**DOI:** 10.1192/j.eurpsy.2024.1584

**Published:** 2024-08-27

**Authors:** N. De Bles, N. Rius Ottenheim, A. van Hemert, E. Giltay

**Affiliations:** LUMC, Leiden, Netherlands

## Abstract

**Introduction:**

Atypical antipsychotic (AAP) drugs are the gold-standard treatment for psychotic patients but are nowadays also widely prescribed among people with other mental disorders. Notwithstanding the benefits of AAP in terms of symptom improvement, there are severe adverse effects including the metabolic syndrome. A novel hypothesis is that part of these undesirable effects of antipsychotics could be mediated by their deleterious effects on the microbiome. This may result in dysbiosis, the disruption of bacterial species of the gut microbiota. Recently, dysbiosis has been linked to poor quality of life, depression and anxiety through the gut-brain axis. Mounting evidence proposes that prebiotic consumption may be helpful in the recovery of dysbiosis, although this effect is unclear among long-term antipsychotic users.

**Objectives:**

The main objective of this study is to assess the potential beneficial effects of the prebiotic Galacto-oligosaccharides (GOS) in combination with 2′-fucosyllactose (2’-FL) on the gut microbiota, by showing a relative increase in Bifidobacteria in fecal samples following intervention. The secondary objective is to assess the effects of GOS on mental wellbeing, sleep, and metabolic parameters. We hypothesize that GOS+2’FL supplementation will improve gut health, mental wellbeing, sleep, and metabolic parameters. Data will be collected 4 weeks prior to the start of the intervention during an observation only phase [t0], at baseline [t1], and after 2 [t2] and 6 [t3] weeks of GOS+2’FL intake. A follow-up will take place at week 10, 4 weeks after the intervention [t4]. Other outcomes that are assessed include the FiberScreen tool, the form of human faeces (Bristol Stool Chart), side effects and the defined daily dosis (DDD) of antipsychotic medication.

**Methods:**

The study is a single-arm pilot study (non-randomized and non-blinded). We aim to include 30 psychiatric patients on long-term atypical antipsychotic use, irrespective of their specific psychiatric disorder, with a BMI > 25 kg/m^2^. Following a run-in period of 4 weeks (no intervention but all other aspects of the study), the participants will consume GOS^plus^ (7.0 g Biotis^TM^GOS + 0.7 g 2’-FL) daily during the first consumption moment of the day (preferably in the morning) for 42 days. The GOS^plus^ powder has a slightly sweet flavour. The primary endpoint is the change in Bifidobacteria in fecal samples from week 0 to week 6.

**Results:**

The study started recruiting participants in October 2023.

**Conclusions:**

Conclusions are expected by the end of 2024.

**Disclosure of Interest:**

None Declared

